# The Impact of Walking Exercises and Resistance Training upon the Walking Distance in Patients with Chronic Lower Limb Ischaemia

**DOI:** 10.1155/2016/7515238

**Published:** 2016-10-19

**Authors:** Maria Szymczak, Grzegorz Oszkinis, Marian Majchrzycki

**Affiliations:** ^1^Clinic of Rheumatology and Rehabilitation, Poznan University of Medical Sciences, Poznań, Poland; ^2^Clinic of General and Vascular Surgery, Poznan University of Medical Sciences, Poznań, Poland

## Abstract

* Objective.* The objective of this paper is to compare the impact of supervised walking and resistance training upon the walking distance in PAD patients.* Materials and Methods.* The examination involved 50 PAD patients at the 2nd stage of the disease according to Fontaine's scale. The participants were randomly allocated to two groups: one exercising on the treadmill (*n* = 24) and one performing resistance exercises of lower limbs (*n* = 26).* Results.* The 12-week program of supervised rehabilitation led to a significant increase in the intermittent claudication distance measured both on the treadmill and during the 6-minute walking test. The group training on the treadmill showed a statistically significant increase of the initial claudication distance (ICD) and the absolute claudication distance (ACD) measured on the treadmill, as well as of ICD and the total walking distance (TWD) measured during the 6-minute walking test. Within the group performing resistance exercises, a statistically significant improvement was observed in the case of parameters measured on the treadmill: ICD and ACD.* Conclusions.* The supervised rehabilitation program, in the form of both walking and resistance exercises, contributes to the increase in the intermittent claudication distance. The results obtained in both groups were similar.

## 1. Introduction

Atherosclerosis (or arteriosclerotic vascular disease; ASVD) is the most frequent cause of lower limb blood-flow disorders. Chronic lower limb ischaemia is a disease manifested through a wide range of clinical symptoms, from the complete absence of symptoms, through intermittent claudication, to critical lower limb ischaemia. Clinical symptoms depend most of all upon the degree of vascular stenosis/occlusion, the location of lesions in particular vascular segments, the degree of advancement of collateral circulation, the general health condition, and the degree of the patient's involvement in carrying out the recommended conservative therapy [1–4].

The most frequent symptom of PAD is intermittent claudication. It manifests itself through the pain in one or both lower limbs during physical exercise, the growing intensity of which forces the patient to stop. The pain in lower limb muscles is connected with temporary ischaemia caused by arterial occlusion or stenosis. The pain is recurrent, invoked by physical effort, and subsides within a few minutes of rest. The location and extent of arteriosclerotic lesions determine the location of painfulness in lower limbs [5].

Literature of the subject describes various algorithms of PAD patients' rehabilitation procedure. The most frequent forms of a walking therapy for chronic intermittent claudication patients include supervised treadmill walking, Nordic walking, and unsupervised walking in the home environment. Additional methods of improving the patient's general fitness include resistance training of lower and/or upper limbs, pedaling a stationary exercise bicycle, or a combination of these methods. Irrespective of its form, rehabilitation should be conducted as a complex, systematic, and continuous therapy, selected individually to account for other coexisting diseases. The main purposes of rehabilitation are [6–8] as follows:

- To extend the walking distance
- To strengthen the lower limb muscles
- To improve the balance and nerve-muscle coordination
- To improve the overall fitness
- To improve hemodynamic parameters of the circulatory and respiratory system
- To improve the quality of life
- To defer invasive treatment or prevent the necessity of a renewed surgical intervention

Each rehabilitation session should be split up into three parts: exercises preparing the muscles for an effort, or the so-called warm-up; the proper training; and stretching exercises. As most patients suffering from lower limb ischaemia are elderly people, often suffering from other cardiovascular diseases, it is of utmost importance to control the pulse during the exercises. At first, the value of the training pulse should not exceed 60% of the value of the maximum pulse [6, 7, 9].

A controversial issue regarding the rehabilitation of PAD patients is the intensity of training. According to TASC II experts (Trans-Atlantic Inter-Society Consensus for the Management of Peripheral Arterial Disease), the rehabilitation of intermittent claudication patients should have the form of supervised walking exercises conducted on a treadmill and should continue over a period of minimum 12 weeks. Exercise sessions should be held three times a week, beginning with 30 minutes of training and then increasing to approximately 1 hour per session. It is recommended that a walking exercise invokes a claudication pain of moderate intensity (below 4 on a 5-point claudication scale). At the onset of such a claudication pain, the patient should stop exercising and take a rest and then undertake another attempt. As the claudication distance increases, the supervisor should increase the speed or grade of the treadmill [1].

To comply with the pain-free model of rehabilitation, the walking exercise should be stopped as soon as the first symptoms of intermittent claudication appear. It is believed that exercises causing a moderate and high intensity of pain are an indicator of an inflammatory reaction. Recurring episodes of ischaemia and reperfusion, occurring during interrupted effort, intensify oxidizing processes and contribute to the damage of vascular endothelium. The pain-free form of exercising consists in interrupting the session as soon as the patient reports a mild claudication pain and then restarting the session as soon as the symptoms of claudication have abated. The effects of pain-free rehabilitation are indicated by the increase of the claudication distance without inducing the unfavorable inflammatory reaction [10–12].

A decrease in pain and an increase in walk distance observed in patients are not the result of a single mechanism but rather a number of various effects of exercise. Mechanisms responsible for the improvement observed in patients are still not entirely clear and are the subject of continuous discussion. It is currently believed that one of the mechanisms responsible for improvement in patients' functioning in response to exercise is an increase in vascular endothelial reactivity. Regular physical exercise increases the synthesis of both nitrogen oxide and prostacyclin, which have a vasodilatory effect. Physical exercise also improves aerobic metabolism and limits anaerobic metabolism in lower limb muscles in patients with intermittent claudication. The improvement in aerobic metabolism observed in patients performing exercise probably results from an increased activity of mitochondrial enzymes, such as cytochrome c oxidase, succinate oxidase, and citrate synthase. The increase in walk distance observed in patients performing exercise is also attributed to positive histological changes in lower limb muscles: greater number of type 2a fibres (fast-twitch muscle fibres with medium oxidative capacity) than type 2b fibres (fast-twitch muscle fibres with low oxidative capacity). Other mechanisms responsible for improvement in patients' functioning in response to exercise include, among others, improvement in the rheological properties of blood, reduction of risk factors of atherosclerosis, and improvement in walking economy. To date, the impact of exercise on the development of collateral circulation in patients with chronic lower limb ischaemia has not been fully confirmed [13–16].

Resistance exercises recommended for PAD patients may be applied along the walking exercises or may be an alternative for patients unable to walk on the treadmill due to, for example, balance disorders or the patient's orthopedic dressing [17, 18]. In patients with intermittent claudication, structural changes within lower limb muscles translate into functional changes, for example, muscle strength and endurance. Muscles which are easily weakened include knee joint flexors, plantar flexors, and dorsal foot muscles. Muscle weakness is often accompanied by lower muscle mass and, in the case of triceps surae, an aggravated function of the fibular nerve [19–21].

Resistance training includes exercises during which muscles generate the strength necessary to overcome a specified resistance. It leads to increasing muscle strength and/or muscle endurance. The most prevalent form of resistance training used in rehabilitation is weight and durability training, which involves the use of small weights and a large number of repetitions [22, 23].

Resistance training for PAD patients is most often carried out in the form of isotonic exercises (building strength when a muscle's length changes) and isokinetic exercises (building strength at a specified steady angular velocity). Resistance in isotonic exercises is provided by dumbbells, rubber bands, weights, or body weight. The starting resistance is set at a level allowing the patient to perform 15 repetitions of each exercise. Isokinetic training involves the use of professional dynamometers (e.g., Biodex, Cybex), which allow for making a motion at a steady angular velocity and adapting the resistance to the musculoskeletal system and the pain, as well as the level of fatigue [6, 23–25].

Irrespective of the type of resistance training, it should be exercised two to three times per week. It is recommended that a session includes three series of 8–12 repetitions of each exercise for each lower limb muscle group. A recess between the series of exercises should be 1-2 minutes [1, 7, 23, 26, 27].

In order to assess the patient's functional abilities and select appropriate rehabilitation parameters, it is necessary to determine the distance of intermittent claudication. Commonly, these parameters are determined during walking tests on a treadmill, the 6-minute walking test, or—less frequently—during pedaling tests on a cycloergometer. The walking tests include protocols with variable load (the so-called Graded Test) and tests during which the load remains constant. During graded tests, the supervisor increases the treadmill velocity or grade. The tests are most commonly conducted at a constant velocity of 3 km/h and the treadmill grade adjustment of 2% every two minutes or 3–5% every five minutes until the grade of 12% is reached. The constant-load walking test usually involves protocols in which the velocity is set at 2.5–3 km/h and the treadmill grade at 8–12%. In the case of a cycloergometer, the test usually begins with a load of 25 watts, which is increased every 2 or 3 minutes by another 25 watts [28–30]. The most recent method of assessing the patient's functional abilities is a test performed with the use of an accelerometer and GPS (Global Positioning System) transmitters, which allow for the detection and recording of motion in space [31, 32].

## 2. Aim

The aim of this study is to compare the impact of supervised walking and resistance training upon the walking distance in PAD patients.

## 3. Material and Methods

### 3.1. Participants

The research involved 27 women and 23 men aged 52–75 suffering from PAD of the 2nd degree according to Fontaine's scale. All participants were patients treated at the Clinic of General and Vascular Surgery of Karol Marcinkowski Medical University in Poznań (The Hospital of Transfiguration of Jesus, 1/2 Długa Street) and the Clinic of General and Vascular Surgery and Angiology of Karol Marcinkowski Medical University in Poznań (L. Bierkowski Ministry of Internal Affairs Public Health Clinic, 34 Dojazd Street). The participants were randomly allocated to two groups: one group performing walking exercises on a treadmill and the other group performing resistance exercises. To confirm the PAD diagnosis and assess the degree of advancement of the disease, the exertional and resting ABI (Ankle Brachial Index) indicator was measured for each patient, separately for the left and right side of the body.

### 3.2. Inclusion Criteria

The following inclusion criteria were adopted: exertional pain in the lower limbs reported by the patient and confirmed in diagnostic walking tests, resting ABI indicator ≤ 0.9, a written consent to participate in the research, and a commitment to participate in at least 80% of the training sessions.

### 3.3. Exclusion Criteria

Conditions regarded as criteria excluding the patient from the research included a symptom-free course of PAD, the technical inability to assess the ABI, the inability to walk on a treadmill, diabetes, joint disorders obstructing motion, the amputation of a lower limb, ulceration of the lower limbs, a stroke suffered within 6 months prior to the research, acute coronary syndrome, coronary artery bypass surgery or endovascular angioplasty of coronary arteries within 12 months prior to the research, a surgical or endovascular intervention in the lower limbs within 12 months prior to the research, aortic aneurysm, advanced kidney or liver failure, the failure of the respiratory or circulatory system, active neoplastic disease, advanced chronic venous insufficiency, Buerger's disease, uncontrolled hypertension, mental disease or dementia obstructing contact with the patient, or simultaneous participation in other clinical researches.

### 3.4. Training Protocol

The research involved a 12-week supervised program of walking on the treadmill and resistance exercises of the lower limbs. Exercise sessions were held twice a week. Each training session lasted 50 minutes. The same training device was used throughout the 12-week period. Exercises were carried out at intervals; the exercise was interrupted as soon as the patient felt a light claudication pain in the lower limbs. A light claudication pain was assessed as the 3rd-degree pain on a 5-point, subjective scale, where 1 indicates no pain, 2 the occurrence of pain, 3 a light pain, 4 a moderate pain, and 5 the maximum intensity of pain. Each training session was split into three parts: warming up exercises (10 minutes), proper training (30–40 minutes), and exercises stretching the lower limb muscles (10 minutes).

The walking exercises were performed on KettlerTrack Motion 7881-300 treadmill (Kettler, Germany). In the early 6 weeks, the exercise sessions lasted 30 minutes each, while later the time was extended to 40 minutes. As soon as the patient suffered a light claudication pain in the lower limbs, the pause button was depressed. After a period of rest, the patient resumed the exercise and the time of the resumed walking was added to the previous result. The resting time between subsequent attempts was decided by the patient. It was recommended that the patient could resume walking only after the claudication pain in the lower limbs had abated completely. The velocity of the treadmill was constant. The initial treadmill grade corresponded to the grade at which the claudication pain appeared in the lower limbs during the diagnostic walking test. If the tolerance to strain was satisfactory, the treadmill grade was increased by 1% every seven days, until the maximum grade of 12% was reached.

Resistance exercises were performed on training devices (Star Trac, USA). They were conducted in the form of stationary training, which allowed for the engagement of various muscle groups: four-headed muscles of the femur (quadriceps femoris), ischiocrural muscles, three-headed muscles of the calf (triceps surae), tibialis anterior muscles, and adductor and abductor muscles of the hip. During the sessions, the patient remained seated. Exercises engaging each group of muscles were performed in three series comprised of 15 repetitions each. The initial resistance was selected individually, so that the patient could easily perform 15 repetitions of each exercise. If the tolerance to strain was satisfactory, the load for each patient was increased by 5% every 7 days. The intervals between subsequent exercises were decided individually by each patient as the shortest time after which no pain was experienced in the subsequent attempt. Before starting an exercise, the training device was adapted individually to the abilities of each patient, taking into account the length of limbs and the location of the turn axis in the knee joint. Resistance exercises were performed slowly, accommodating the full scope of motion, and were synchronized with respiration (exhalation during resistance).

### 3.5. Walking Tests

Up to seven days prior to the research, as well as up to seven days after completing the program, the participants were subjected to the following tests:

- Pain-free walking distance on the treadmill (initial claudication distance, ICD)
- Absolute claudication distance (ACD) on the treadmill
- Initial claudication distance (ICD) in the 6-minute walking test
- Total walking distance (TWD) in the 6-minute walking test
- The number of intervals in the 6-minute walking test.

The measurement of the claudication distance on the treadmill was taken with the use of Gardener's protocol with a gradually increased grading. The treadmill velocity was 3.2 km/h and remained constant. During the first two minutes, patients walked on a flat surface, without any grading of the treadmill. The grading was increased by 2% every two minutes, until the maximum grading of 12% was reached. The initial claudication distance was the part of the distance covered until the first symptoms of claudication pain appeared in the lower limbs. The absolute claudication distance was the part of the distance covered until the patient had to stop walking as a result of a strong pain and muscle cramps in the lower limbs.

The 6-minute walking test was carried out on a marked square of 50 m circumference. The test involved the patient's attempt to cover the maximum distance within 6 minutes. The patient was allowed to walk at his desired speed, which excluded trotting and running. The patient was allowed to stop and rest at any time, and every such situation was recorded.

### 3.6. ABI

The reading of ABI was taken with the use of Doppler SmartDop 45 (Hadeco, Japan) with an ultrasound probe of a frequency of 8 MH. The measurements were carried out in the prone position after a 10-minute period of adaptation (resting ABI) and after a diagnostic walking test on the treadmill (exertional ABI).

## 4. Statistics

The statistical analysis was performed with the use of Statistica 12.5 software. In order to assess the normality of distribution of the analyzed parameters, Shapiro-Wilk test was applied. For parameters that did not show the normality of distribution, the descriptive statistics were presented as a median with range. Parameters analyzed as dependent variables were compared with the use of Wilcoxon's test, while those analyzed as independent variables were compared with the use of Mann–Whitney test.

## 5. Results

Population data concerning the participants allocated to the two groups were comparable as regards the gender, age, and duration of the disease (*p* > 0.05 in the Mann–Whitney test and Person's chi-square test). In the case of walking tests, the results of examinations conducted prior to the start of the therapy indicated no differences between the compared groups. The statistical analysis of ABI parameters, compared prior to joining the rehabilitation program, indicated a statistically significant difference between the groups as regards the resting ABI (a lower value for the treadmill walking group). No statistically significant difference was observed for the remaining parameters (Table 1).

The 12-week program of supervised rehabilitation resulted in a significant increase in the claudication distance measured both on the treadmill and during the 6-minute walking test. The increase was statistically highly significant for both patients exercising on the treadmill and those performing resistance exercises.

In the case of patients exercising on the treadmill, a statistically significant improvement was observed for four out of five parameters of the walking tests (Table 1). In the case of patients performing resistance exercises, two out of five parameters of the walking tests improved in a statistically significant way (Table 1). In both groups, no changes were observed in the number of intervals the patient needed to complete the 6-minute walking test, as well as no changes were observed in the ABI indicator.

The research showed no statistically significant change between the groups as regards the improvement of the parameters obtained in the walking tests (Table 2).

It was determined how the initial gait parameters correlate with the changes in parameters after the treatment. The research presents correlations between the values of parameters obtained in walking tests before the treatment and changes in parameters after the treatment. In the case of most of the parameters analyzed no correlation was found between the initial values (stage of lower limb ischaemia) and the extent of improvement observed after the exercise programme. For both groups, a statistically significant result was observed for only one parameter, namely, the number of rest periods needed to complete a 6-minute walking test (Table 3). It may be assumed that other factors influence the extent of improvement observed after the treatment, which should be the subject of further study.

## 6. Discussion

Randomized, prospective tests indicated the effectiveness of the 12-week supervised rehabilitation program for PAD patients. The effectiveness of training indicated by the improvement of the intermittent claudication distance was similar for both randomly allocated groups of participants: one performing walking exercises on the treadmill and the other performing resistance exercises. This is the first prospective research in Poland which indicates that it is possible to use both rehabilitation methods as alternatives.

The results of this research are unanimous with the results obtained by Ritti-Dias et al., who compared the results of a 12-week program of resistance exercises and walking exercises performed twice a week. The walking distance was also measured with a graded protocol on a treadmill. The intensity of exercises was established on the level of 11–13 on Borg's scale, which corresponded to level 3 on a 5-point scale used in our research. Ritti-Dias observed a significant improvement of ICD and ACD measured on the treadmill in both groups of participants. The results obtained in both groups did not differ in a statistically significant way. Measured in meters, ICD in the treadmill walking group grew from 342 ± 182 to 469 ± 237, while ACD grew from 572 ± 231 to 721 ± 287 (*p* < 0.05). The pain-free distance and ACD in the group performing resistance exercises grew, respectively, from 335 ± 224 to 504 ± 276 and from 618 ± 282 to 775 ± 334 (*p* < 0.05). The only difference observed by Ritti-Dias et al. was a lower level of pain experienced by patients during the walking exercises compared with those performing resistance exercises [26].

A similar impact of walking exercises and resistance training upon the walking distance was also indicated by Cucato et al. Their research involved a 12-week program of exercises with the intensity corresponding to levels 11–13 on Borg's scale. The improvement of walking parameters in both groups was similar. In the treadmill walking group, ICD measured in meters grew from 342 ± 182 to 469 ± 237, while ADC grew from 572 ± 231 to 721 ± 289 (*p* < 0.01). In the group performing resistance exercises, ICD grew from 358 ± 224 to 504 ± 276, while ACD grew from 618 ± 282 to 775 ± 334 (*p* < 0.01). Measurements were taken on a treadmill with a graded protocol [33].

The results of our research are also unanimous with the results obtained by McDermott et al., who allocated 156 PAD patients to three groups: group 1 performing walking exercises on the treadmill, group 2 performing resistance exercises, and the control group which only received dietary recommendations. Compared with the control group, ACD measured on the treadmill improved in a statistically significant way in both the group performing walking exercises and the group performing resistance exercises. Similar to our research, the differences were observed in the case of the 6-minute walking test. The improvement of TWD was observed only in the group performing on the treadmill [34].

The results of our research are not compatible with the results obtained by Hiatt et al., whose results indicated the advantage of walking exercises over resistance exercises in improving the pain-free time. In the treadmill walking group, an increase of 74 ± 58% in the maximum claudication time was observed after 12 weeks in comparison with the initial values. Moreover, VO2 max (maximal oxygen uptake) and the pain-free time also increased in a statistically significant way. In the group performing resistance exercises, the maximum walking time increased merely by 36 ± 48%, while VO2 max and the pain-free time did not change in a statistically significant way. It is worth noting that, in Hiatt et al.'s research, five out of nine participants were active smokers. In our research, smoking was reported by nine out of 26 participants. The unfavorable impact of smoking on rehabilitation effects is confirmed by ABI measurements. The participants in Hiatt et al.'s research, notwithstanding a shorter duration of their illness (2 ± 1 versus 7 years), indicated a lower ABI than the participants in our research. Another element worthy of mentioning is the methodological differences regarding resistance training. In our research, the initial resistance was selected individually, so that the patient would be able to perform 15 repetitions of each exercise. In Hiatt et al.'s research, the initial resistance was set at the level at which a light pain appeared as early as after 6 repetitions. It may be inferred that the weight and durability training selected for our research turned out to be more effective than the training selected by Hiatt et al. [35].

## 7. Conclusions

1. Both walking exercises and resistance training contribute to a significant improvement of the claudication distance in PAD patients.
2. In the applied model of rehabilitation, the observed results were similar for both study groups in relation to most analyzed walking parameters.
3. Resistance training may provide an alternative for treadmill walking exercises for PAD patients.

## Figures and Tables

**Table 1:** Differences between data medians in patients performing walking exercises and resistance training prior to the therapy (where *p* = the level of significance).

Variable	Walking exercises				Resistance training			
	Before	After	% of change	*p* value	Before	After	% of change	*p* value
Initial claudication distance [m] (treadmill)	78.3 (±64.1)	168.5 (±113.2)	115%	**<0.001**	125 (±134.7)	186.7 (±211.9)	49%	**0.027**
Absolute claudication distance [m] (treadmill)	172.3 (±137.8)	318.3 (±230.8)	85%	**0.001**	254.3 (±223.3)	402.7 (±501.6)	58%	**0.033**
Initial claudication distance [m] (6-minute walking test)	108.8 (±85.3)	178.5 (±111.6)	64%	**0.001**	135.6 (±123.6)	160.4 (±130.6)	18%	0.204
Number of intervals (6-minute walking test)	1.4 (±1.5)	1.1 (±1.4)	−21%	0.251	0.9 (±1)	0.6 (±0.9)	−33%	0.103
Total walking distance [m] (6-minute walking test)	305.1 (±78.1)	346.9 (±888)	14%	**0.004**	322.7 (±71.4)	349.8 (±94.9)	8%	0.076
Resting ABI right side	0.69 (±0.14)	0.68 (±0.15)	−1%	0.624	0.65 (±0.14)	0.66 (±0.13)	2%	0.671
Resting ABI left side	0.65 (±0.14)	0.67 (±0.14)	3%	0.234	0.75 (±0.12)	0.73 (±0.14)	−3%	0.441
Exertional ABI right side	0.6 (±0.15)	0.58 (±0.14)	−3%	0.413	0.53 (±0.11)	0.56 (±0.12)	6%	0.698
Exertional ABI left side	0.57 (±0.13)	0.58 (±0.13)	2%	0.309	0.65 (±0.14)	0.63 (±0.15)	−3%	0.476

ABI: Ankle Branchial Index.

**Table 2:** Posttherapy comparison of measured differences between study groups.

Variable	Walking exercises	Resistance training
Initial claudication distance [m] (treadmill)	90.2 (±115.1)	61.7 (±169.9)
Absolute claudication distance [m] (treadmill)	145 (±232.5)	148.3 (±446.9)
Initial claudication distance [m] (6-minute walking test)	69.6 (±107.6)	18.12 (±93.6)
Number of intervals (6-minute walking test)	−0.3 (±1.2)	−0.33 (±0.9)
Total walking distance [m] (6-minute walking test)	41.8 (±68.8)	27.1 (±65.9)
Resting ABI right side	−0.004 (±0.177)	0.006 (±0.041)
Resting ABI left side	0.02 (±0.115)	−0.02 (±0.095)
Exertional ABI right side	−0.02 (±0.201)	0.022 (±0.078)
Exertional ABI left side	0.007 (±0.082)	−0.014 (±0.078)

ABI: Ankle Branchial Index.

**Table 3:** Correlation between the values of parameters obtained in walking tests before the treatment and the changes in parameters after the treatment.

Variable	Walking exercises		Resistance training	
	Spearman's rank correlation coefficient	*p* value	Spearman's rank correlation coefficient	*p* value
Initial claudication distance [m] (treadmill)	−0,259	0,202	−0,343	0,100
Absolute claudication distance [m] (treadmill)	−0,094	0,648	−0,251	0,237
Initial claudication distance [m] (6-minute walking test)	−0,163	0,428	−0,384	0,064
Number of intervals (6-minute walking test)	−0,462	**0,018**	−0,523	**0,009**
Total walking distance [m] (6-minute walking test)	−0,233	0,252	−0,117	0,588
